# Predicted Auxiliary Navigation Mechanism of Peritrichously Flagellated Chemotactic Bacteria

**DOI:** 10.1371/journal.pcbi.1000717

**Published:** 2010-03-19

**Authors:** Nikita Vladimirov, Dirk Lebiedz, Victor Sourjik

**Affiliations:** 1 Interdisziplinäres Zentrum für Wissenschaftliches Rechnen (IWR) der Universität Heidelberg, Heidelberg, Germany; 2 Zentrum für Biosystemanalyse (ZBSA) der Universität Freiburg, Freiburg, Germany; 3 Zentrum für Molekulare Biologie der Universität Heidelberg, DKFZ-ZMBH Alliance, Heidelberg, Germany; University of Illinois at Urbana-Champaign, United States of America

## Abstract

Chemotactic movement of *Escherichia coli* is one of the most thoroughly studied paradigms of simple behavior. Due to significant competitive advantage conferred by chemotaxis and to high evolution rates in bacteria, the chemotaxis system is expected to be strongly optimized. Bacteria follow gradients by performing temporal comparisons of chemoeffector concentrations along their runs, a strategy which is most efficient given their size and swimming speed. Concentration differences are detected by a sensory system and transmitted to modulate rotation of flagellar motors, decreasing the probability of a tumble and reorientation if the perceived concentration change during a run is positive. Such regulation of tumble probability is of itself sufficient to explain chemotactic drift of a population up the gradient, and is commonly assumed to be the only navigation mechanism of chemotactic *E. coli*. Here we use computer simulations to predict existence of an additional mechanism of gradient navigation in *E. coli*. Based on the experimentally observed dependence of cell tumbling angle on the number of switching motors, we suggest that not only the tumbling probability but also the degree of reorientation during a tumble depend on the swimming direction along the gradient. Although the difference in mean tumbling angles up and down the gradient predicted by our model is small, it results in a dramatic enhancement of the cellular drift velocity along the gradient. We thus demonstrate a new level of optimization in *E. coli* chemotaxis, which arises from the switching of several flagellar motors and a resulting fine tuning of tumbling angle. Similar strategy is likely to be used by other peritrichously flagellated bacteria, and indicates yet another level of evolutionary development of bacterial chemotaxis.

## Author Summary

Chemotaxis of bacteria plays an important role in their life, providing them with the ability to actively search for an optimal growth environment. The chemotaxis system is supposed to be highly optimized, because on the evolutionary time scale even a modest enhancement of its efficiency can give cells a large competitive advantage. For a long time it was believed that the only navigation mechanism of bacteria is increasing the run length toward the preferred direction. The tumble was assumed to be a purely random change of direction between runs. We analysed recently published experimental data that demonstrate a dependence of tumbling angle on the number of CW-switched motors. We introduced such a dependence into our model of chemotactic *E. coli*, and simulated it under different conditions. Our simulations show that this dependence is an important additional mechanism of bacterial navigation, which was previously unrecognized because it lays below the experimental errors of conventional single-cell tracking. We show that such a fine tuning of tumbling significantly improves efficiency of chemotaxis, and represents a new level of evolutionary optimization of bacteria.

## Introduction

Many motile unicellular organisms are known to direct their movement in gradients of specific chemical substances – the process called chemotaxis. Chemotaxis plays an important role in the microbial population dynamics with chemotactic bacteria in a nonmixed environment – that is in presence of nutrient gradients – having significant growth advantage [1]–[4]. Modeling of microbial population dynamics indicates that motility and chemotactic ability can be as important for evolutionary competition as cell growth rate [5],[6]. The chemotaxis system is thus expected to be highly optimized, as has been indeed suggested by several studies [7]–[10].

The best example of such optimization is bacterial chemotaxis strategy itself. While eukaryotic cells are able to sense the gradients by direct comparison of concentrations at the opposite sides of the cell [11], bacteria like *E. coli* employ temporal comparisons along their runs [12]. Theoretical analysis suggested that such strategy is superior to direct spatial comparisons for objects of bacterial size and swimming speed [7]. Adapted *E. coli* has two swimming modes: runs, which are periods of long straight swimming, and tumbles, when bacterium stops and changes its orientation. The runs of a swimming bacterium are interrupted by tumbles which abruptly change the swimming direction. For cells swimming up an attractant gradient, the runs become longer due to suppression of tumbles, and the cell population migrates up the gradient. The frequency of tumbles is controlled by the chemotaxis network through switching of individual motors. During a run, flagellar motors rotate counter-clockwise (CCW) causing flagella to form a bundle, whereas switching of one or several flagellar motors to clockwise (CW) rotation breaks up the bundle and initiates a tumble. The direction of motor rotation depends on the concentration of phosphorylated CheY molecules, which bind to the motor and switch its direction in a highly cooperative mode. The CheY phosphorylation is controlled by the histidine kinase CheA, which forms sensory clusters together with transmembrane receptors and the adaptor CheW. Each receptor can be either active or inactive, depending on ligand binding and on the methylation level. The active receptor activates CheA, eliciting downstream phosphorylation of the response regulator CheY. Phosphorylated CheY (CheYp) is dephosphorylated by CheZ. Receptors can be methylated by the methyltransferase CheR and demethylated by the methylesterase CheB. Methylation regulates the receptor activity. Because the reaction of receptor methylation is much slower than the initial response, methylation provides chemical ‘memory’, which allows the cell to compare the current ligand concentration with the recent past.

Early single-cell tracking experiments reported no dependence of the tumbling angle, i.e. turning angle between consequent runs, on the direction of the gradient and the inclination of a run [12], and it was thus presumed to be random in subsequent modeling of bacterial chemotaxis. However, in recent study that used high-resolution fluorescence video microscopy [13], it was shown that the cell turning angle depends on the number of CW-rotating filaments involved in the tumble, and thereby the turning angle rises proportionally to the number of motors that switched to CW rotation. Because the CW switch probability is set by the chemotaxis system dependent on the cellular swimming direction along the gradient, the tumbling angle can be expected to depend on the swimming direction, too. If the cell swims up a gradient of attractant, the probability of CW rotation is smaller, and fewer motors are likely to change directions. Therefore, even if the cell makes a tumble, the tumbling angle should be small. When the cell swims down the gradient of attractant, the probability of CW rotation is higher and more motors are likely to change directions during a tumble, with the consequence that the tumbling angles will be larger.

The goal of this study was thus to investigate the magnitude of the tumbling angle dependence on the swimming direction and the effect of such dependence on the chemotactic efficiency. We introduced dependence of the turning angle on the number of CW-rotating motors in a recently constructed hybrid model of chemotactic *E. coli*, RapidCell simulator [14]. Our simulations demonstrate that although the estimated difference of tumbling angles up and down the gradient is only few degrees, even such a small difference significantly improves the chemotactic efficiency of *E. coli*. We thus suggest that tuning of tumbling angle depending on swimming direction serves as an additional navigation mechanism for *E. coli* and other peritrichously flagellated bacteria with similar chemotaxis behavior.

## Results/Discussion

### Dependence of tumbling angle on the number of CW-rotating motors

**Figure 1:**
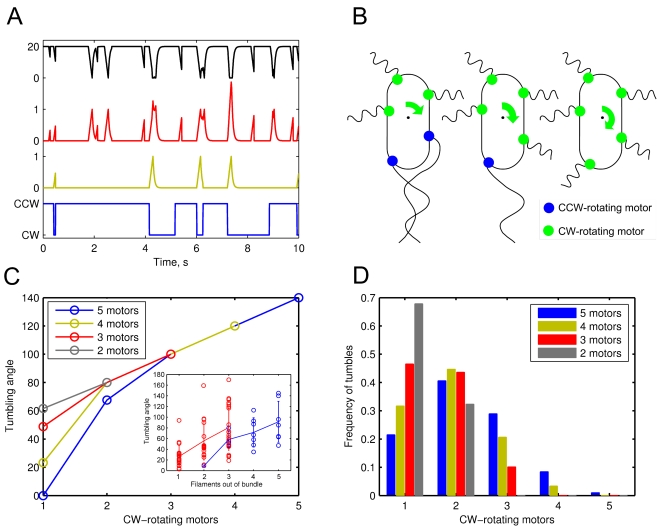
Anisotropic model of *E. coli* tumbling. (A) The output series for a single swimming cell (from bottom to top): switching of a single motor (blue), its distortion 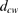 (green), the sum of distortions of 3 motors 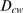 (red), the resulting falls of swimming speed during tumbles (black). (B) The schematic illustration of tumbling angle (green arrow) dependence on the number of CW-rotating motors (green circles). (C) Anisotropic model of tumbling. The tumbling angle 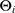 at different number of CW-rotating motors 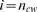. *Inset*. Experimental data sets reproduced from Fig. 12 of [13]. Solid lines show means, errorbars show standard deviations, circles correspond to individual tumbles. Color code of the inset is the same as in the main panel. (D) Frequencies 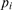 of tumbles which involve 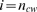 CW-rotating motors out of the total number of motors 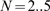.

The tumbling angle dependence on the number of switching motors was investigated by extending the recently published hybrid model of chemotactic *E. coli* [14]. First, a more detailed model of tumbling was developed to bring the model in a closer agreement with the tracking experiments of [12]. While previous version of the model relied on a simple voting model of tumbling, which started the tumble as soon as the majority of motors rotate CW, our new model takes into account the duration of CW-rotation of every motor (Fig. 1A). The complex hydrodynamics of multiple flagella is described in simplified form, through a distortion factor which is a function of 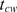 of each motor (see Methods). Despite this simplification, the simulated swimming of *E. coli* is in a very good agreement with the original tracking experiments [12]. The model realistically reproduces nearly all data provided by tracking experiments: mean cellular speed, run times, tumbling angles (Tab. 1), as well as individual motor switching and graduate recovery of cellular speed after a tumble.

**Table 1:** Comparison of the RapidCell output and the tracking data from (Berg and Brown, 1972).

Parameter	Isotropic model	Anisotropic model	Experiment
Tumbling angle, control (  )	67.5	67.5	68
Run length, control (s)	0.81 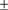 0.63	0.81 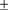 0.63	0.86 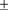 1.18
Run length, gradient (s)	0.89 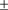 0.77	0.92 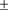 0.86	0.90 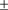 1.56
Run length, up gradient (s)	0.93 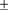 0.83	0.98 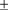 0.95	1.07 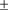 1.80
Run length, down gradient (s)	0.83 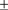 0.69	0.86 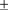 0.75	0.80 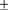 1.38
Swimming speed, control ( 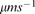 )	17 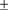 5.4	17 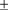 5.4	14.2 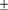 3.4
Drift velocity, control ( 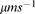 )	0.36 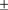 0.03	0.39 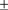 0.03	–
Drift velocity, gradient ( 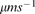 )	0.92	1.40	0.90

The model parameters are as in Tab. 2, the number of motors 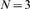, the gradient is N1. Values are estimated from 1000 cells simulated for 500 s. Controls correspond to a ligand-free medium. Means and std (where relevant) are shown.

**Table 2:** Parameters used in *E. coli* model.

Parameter	Value	Reference
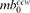	0.65	Steady-state CCW motor bias [30],[31]
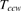	1.33 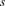	Av. CCW rotation time of a motor at resting state [34]
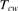	0.71 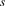	Av. CW rotation time, given that 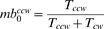
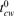	0.15 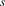	Max. time the flagellum rotates CW in semicoiled form [13]
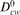	1.0	Threshold of total distortion to initiate a tumble [13]
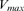	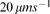	Maximum swimming speed [12],[35]
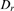	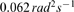	Rotational diffusion coefficient [17]
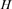	10.3	Hill coefficient of motor response to [CheYp] [30]
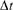	0.01 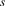	Time step in simulations (this work)
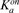	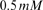	Diss. constant of Tar to MeAsp [22]
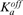	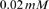	Diss. constant of Tar to MeAsp [22]
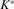	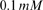	Geometric mean of 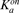 and 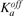

Second, we introduced a dependence of tumbling angle on the number of CW-rotating motors that cause the tumble (Fig. 1B). This was done by fitting the experimental data of [13] with a realistic choice of discrete tumbling angles at each number of CW-switched motors (Fig. 1C). To ensure consistency with experimental data, we further assumed dependence of tumbling angle on the total number of motors. This model was called anisotropic, and it was compared to a conventional model of isotropic tumble, which chooses the tumbling angle stochastically. In simulations without a gradient, both models produce equal cellular drift velocities, with the accuracy of estimation error. To keep the mean angles of both models consistent, we defined the frequencies of the discrete angles in the anisotropic model as shown in Fig. 1D.

### Dependence of tumbling angle on swimming direction

The model of swimming proposed here allows tumbling with variable number of motors, as soon as the sum of their CW-rotation times exceeds 0.15 s threshold needed for tumbling (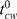). A cell swimming down the gradient will sooner reach the threshold, because each motor has higher probability of switching to CW. As a first consequence, the average run down the gradient will be shorter. As a second consequence of higher switching probability, the average number of motors that switch CW during that tumbling period will be higher than in case of up-gradient swimming. For example, cells with 3 motors when swimming down the gradient N1 tumble with 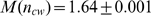 motors while up the gradient with 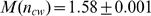 motors (mean
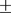
s.e.m.).

**Figure 2:**
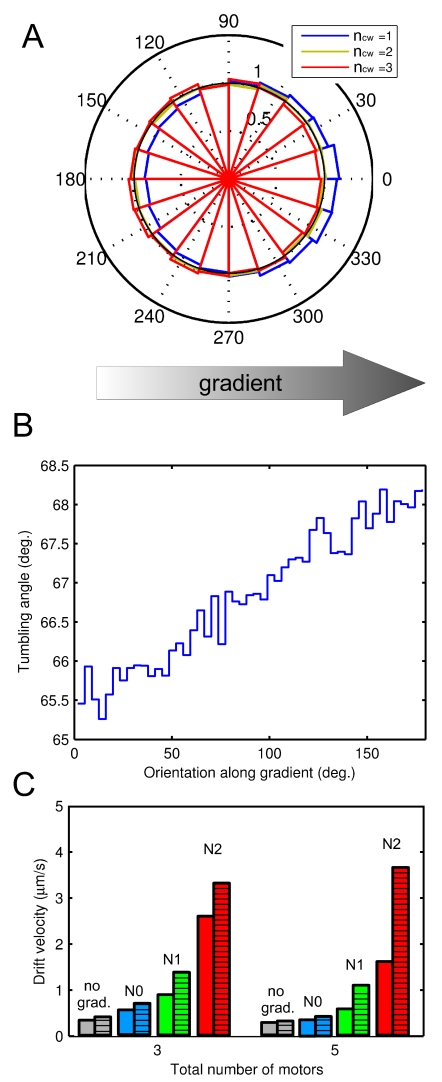
Behavior of cells with anisotropic tumbling model. (A) Distribution of cellular orientations prior to tumbles. The tumbling events are divided into 3 groups, by the number of CW-rotating motors involved in a tumble. The rose histograms are normalized by the number of counts. The inner black circle shows unbiased (isotropic) distribution as a reference. Cell orientation is given relative to the gradient. The gradient steepness is N1. (B) Average tumbling angle as a function of orientation along the gradient prior to tumbles. (C) Chemotactic drift velocity of cells in gradients of different steepness. Bars show the drift velocities of cells with 3 motors (left group) or 5 motors (right group) in the medium without a gradient (gray), in gradient N0 (blue), N1 (green) and N2 (red). Left bars show the isotropic model, right (hatched) bars – anisotropic model of tumbling. In the absence of gradient, the difference is within the error of estimation. Standard error of the mean is about 0.03. Cells in (A) and (B) have 3 motors, other parameters are as described in Tab. 2. The number of simulated cells is 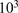 in each case.

Therefore, the tumbling angles for anisotropic model depend on the swimming direction prior to tumbles (Fig. 2A). This dependence naturally arises from the dependence of tumbling angle on the number of CW-rotating motors. The simulated cells which turned with the smallest 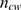 were swimming in slightly skewed directions up the gradient before the tumble, whereas the cells which turned with the highest 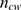 were swimming with even smaller skew down the gradient before the tumble. A more detailed analysis shows that the total angular difference between tumbling angles that correspond to the movement up and down a gradient is only about 3^
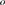
^ (Fig. 2B). Such a small difference is within the error of the early tracking experiments, about 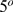 [15], which explains why it remained undetected.

### Effect of anisotropic model on cell drift velocity

Despite such a small difference of mean angles, it can significantly increase the chemotactic performance, with the mean drift velocity being up to two times higher for anisotropically tumbling cells (Fig. 2C). The positive effect of anisotropic tumble becomes more visible in steeper gradients and for higher number of motors, which suggests that highly flagellated cells can adjust their tumbling angle more precisely.

In the case of 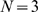 motors and moderate gradient (N1), the mean tumbling angle is 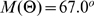. This value is only 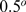 smaller than the angle in ligand-free simulations, so the increase of the drift velocity in the anisotropic model cannot be attributed to the change of the total mean tumbling angle. The mean tumbling angle up the gradient 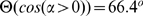, while down the gradient it is 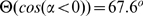. Therefore, the 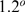 difference in mean tumbling angles causes a 52% increase in the population drift velocity, from 0.92 to 1.4 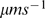 (Fig. 2C).

### Dependence of anisotropic model effect on the magnitude of angle adjustment and on rotational diffusion

**Figure 3:**
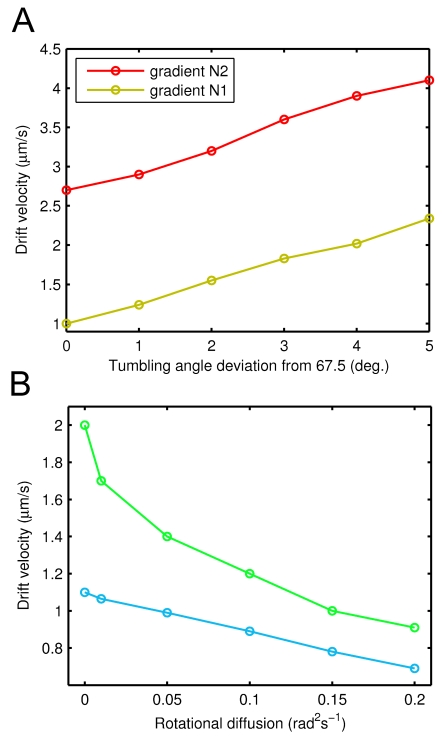
Effects of tumbling angle adjustment and rotational diffusion on chemotactic efficiency. (A) Dependence of chemotactic drift velocity on fixed tumbling angle deviation 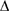 in a simplified tumbling model. The cells swimming up the gradient tumble with a smaller angle 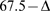, while cells swimming down the gradient tumble with higher angle 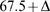. Cells with 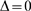 tumble with a fixed angle 67.5*^o^*, i.e. isotropically. (B) Dependence of chemotactic drift on rotational diffusion coefficient for cells with isotropic (blue) and anisotropic (green) models of tumbling. The number of simulated cells is 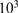 in each case, the gradient is N1. Cells in (A) and (B) have 3 motors, other parameters are as described in Tab. 2.

As a control, we simulated chemotactic cells that tumble with a constant angle (67.5 deg.), and compared them to cells that tumble with slightly smaller angle (67.5−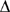), when they swim up the gradient, and with slightly higher angle (67.5+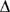), when they swim down the gradient. Here, the 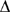 was a constant parameter changed from 1 to 5 deg. A difference of 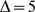 degrees increased the drift velocity by about 100% in the gradient N1, and by 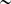 50% in the gradient N2 (Fig. 3A). This confirms that the observed increase in drift velocity shown in Fig. 2C is due to small changes in tumbling angles of up- and down-swimming cells, and does not arise from model-specific parameters.

Bacterial movement in gradients is further affected by the Brownian motion for both isotropic and anisotropic tumbling models (Fig. 3B). In our simulations we used 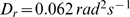 (Tab. 1). At lower coefficients of rotational diffusion, both models demonstrate better chemotaxis, and the advantage of the anisotropic tumbling is most pronounced, which is due to lower noise factor arising from rotational diffusion [16]. Since rotational diffusion depends on the cells size, flagellar length, media viscosity and temperature [17],[18], predicted effects of anisotropic tumbling can be even more pronounced for other bacteria or under different environmental conditions.

### Conclusions

**Figure 4:**
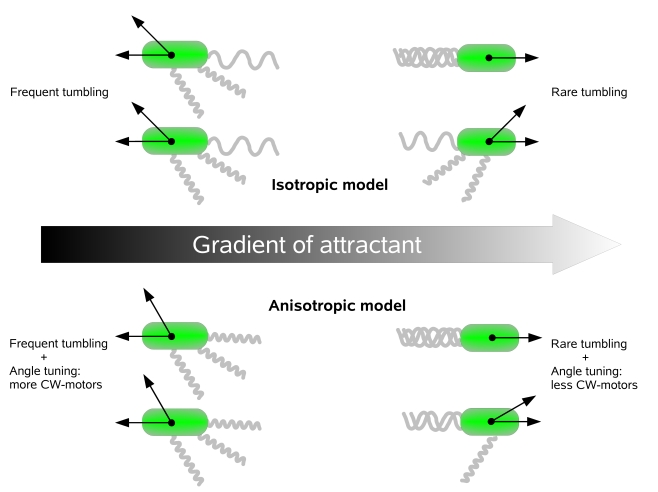
Enhancement of chemotactic efficiency by anisotropic tumbling. In the isotropic model (top), cells have lower CW bias and tumble less frequently up the gradient, but their average tumbling angle is the same in all directions. In the anisotropic model (bottom), the same lowering of CW motor bias additionally leads to the reduction of tumbling angles below average for cells swimming up the gradient. Cells swimming down the gradient have tumbling angles larger than the average. Directional dependence of the tumbling angle enhances average drift up the gradient. The difference of tumbling angles is exaggerated for illustration purposes.

Taken together, our results suggest that in addition to extending the run length while swimming up the gradient, *E. coli* uses an auxiliary mechanism of tumbling angle tuning according to the swimming direction. This fine tuning of tumble is mediated by the same adjustment of tumbling frequency that underlies the conventional chemotaxis strategy of *E. coli* (Fig. 4). Since both navigation mechanisms arise from the same basic mechanism of altered motor switching, evolutionary optimization of the basic mechanism depends on both the effect from the tumble frequency and the number of flagella that reverse per tumble. The previously unrecognized mechanism shown here is expected to be shared by other peritrichously flagellated bacteria with similar chemotactic behavior, and it seems to represent yet another level of evolutionary optimization of the chemotaxis system.

## Methods

### Model of chemotaxis signaling network

We applied the recently proposed Monod-Wyman-Changeux (MWC) model for mixed receptor clusters [19],[20], which accounts for the observed experimental dose-response curves of adapted cells measured by *in vivo* FRET experiments [19],[21], as shown in [20],[22],[23]. According to the MWC model, an individual receptor homodimer is described as a two-state receptor, being either ‘on’ or ‘off’, with the free energy being a function of methylation level 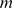 and ligand concentration 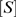


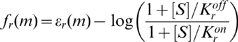
(1)where 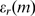 is the ‘offset energy’, and 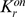, 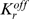 are the dissociation constants for the ligand in the ‘on’ and ‘off’ state, respectively. Groups of receptors form larger sensory complexes, or signaling teams, with all receptors in a team being either ‘on’ or ‘off’ together. The teams are composed of mixtures of Tar 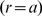 and Tsr 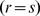 receptors, and the total free energy of the team is given by
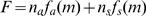
(2)The probability (A) that a team will be active is a function of its free energy
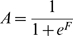
(3)


The adaptation is modeled according to the mean-field theory [24],[25], assuming that the CheB demethylates only active receptors, CheR methylates only inactive receptors, and both enzymes work at saturation
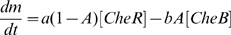
(4)This equation implies that both enzymes work in the zero-order regime. The linear products 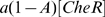 and (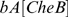) mean that a bound CheR (CheB) can only act if the receptor team is inactive (active), with probability 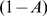 and 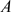, respectively.

The average methylation level 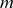 is assumed to be a continuously changing variable within the interval 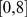, with linear interpolation between the key offset energies, 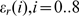, as suggested in [25],[26]. The ODE for methylation (Eqn. 4) is integrated using the explicit Euler method to ensure high computational speed of the program, while the time step is chosen as 0.01 s to keep the simulation error low.

The details of network model were previously described in [14]. CheA kinase activity is assumed to be equal to the activity of the receptor complex 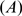. The rate of phosphotransfer from active CheA to CheY is much faster than the rate of CheA autophosphorylation [9],[27]. Therefore, the relative concentration of CheYp is obtained as a function of active CheA from the steady-state equation
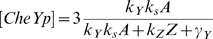
(5)where 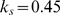 is a scaling coefficient, 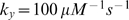, 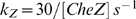, 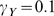 are the rate constants according to [9],[28],[29].

The relative concentration of CheYp is converted into the CCW-motor bias using a Hill function [30]:
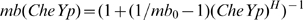
(6)where 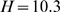 [30], 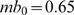 [30],[31].

### Model of bacterial swimming

To simulate the experimentally observed hydrodynamics of bacterial swimming and tumbling [13],[32] in simple terms, we introduce a *distortion* factor 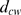 which reflects how one CW-rotating flagellum influences the cellular speed and angular deviation
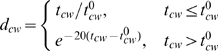
(7)This functional form implies that the distortion rises proportionally to the CW rotation time 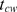 as long as it is below the threshold 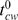 (the first period). After this threshold is reached, the distortion exponentially decays (the second period). The first period corresponds to unwinding of a flagellum from the bundle and its rotation in the right-handed semicoiled form, which initiates a tumble. In the second period, when the flagellum rotates CW longer than the threshold time, a rapid transformation from semicoiled to curly 1 form occurs, and the flagellum twists around the bundle during the new run, due to high flexibility of the latter form [32].

The influence of several simultaneously CW-rotating motors is assumed to be proportional to the sum of their distortion factors
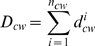
(8)This implies that the tumble can occur if a single motor rotates CW for at least 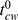 period, or if two or more motors rotate CW together for a shorter time. Formally, a tumble occurs when 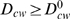, where 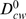 is a threshold value. In principle, the threshold depends on the total number of motors: the larger 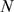, the higher 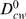 is required to generate a tumble. This is consistent with experimental data of [13], Fig. 12 therein, where switching of 1 motor is sufficient for a tumble at 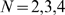, but for 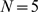 at least 2 motors are necessary for a tumble. However, we keep the same 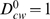 for 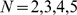 for simplicity, to avoid additional arbitrarily chosen thresholds. The simulated run lengths in a ligand-free medium have distribution close to exponential.

The cellular swimming speed depends on the distortion in a piece-wise linear form
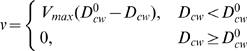
(9)In our model, we considered only ‘complete’ tumbles, which occur when 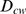 reaches 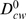 and the swimming speed falls to zero: at this time point the cell instantly changes its orientation by the tumbling angle 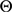, which is determined by two alternative models, isotropic and anisotropic. For simplicity, we assumed that if the distortion 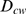 does not reach 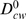, it causes only a drop of speed, without a change of the swimming direction.

During a run, the direction of cellular swimming is affected by the rotational diffusion [12],[17]. After each time step, the swimming direction is changed by adding a stochastic component with normal distribution 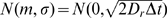, where the diffusion coefficient 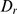 equals 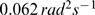 [17].

#### Isotropic tumbling

The tumbling angle 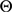 is distributed according to the continuous probability density function 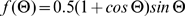, 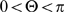, as suggested in [33]. The mean 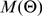 of this angle distribution, 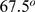, is close to experimental measurement of 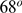 [12], and shapes of the simulated and experimental distributions are simular. The angle distribution does not depend on any external factors.

#### Anisotropic tumbling

The tumbling angle 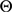 is determined by number of CW-rotating motors 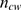 involved in the tumble, and the total number of motors 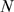. For each pair of 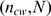, we simulated the cell swimming in a ligand-free medium and calculated the frequency 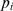 of the tumbles which are caused by 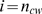 CW-rotating motors. Using the frequency 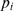, we chose the turning angle 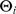 close to the experimental values [13], while keeping the average turning angle constant in all models,
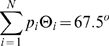
(10)Note that here tumbling angles are discrete, as opposed to the continuous probability density function of isotropic tumble.

The program RapidCell is available at www.rapidcell.vladimirov.de.

### Constant-activity gradient

In order to measure the chemotactic efficiency accurately and to avoid the effects of receptors saturation, we simulated the cells in an artificial constant-activity gradient, which ensures a constant chemotactic response CheYp and a constant cell drift velocity over a wide range of ligand concentrations, in contrast to commonly used Gaussian and linear gradients [14]. Drift velocity in constant-activity gradient was measured by a linear fit of 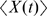 in the time interval from 200 to 500 s. The constant-activity gradient has the following form:
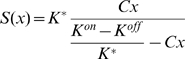
(11)where 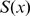 is the ligand concentration in position 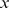, and 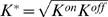 is the geometric mean of Tar methyl-aspartate dissociation constants. Here 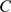 is a free parameter which determines the steepness of the gradient, and thereby the drift velocity of cells up the gradient. We compare the drift velocities in three constant-activity gradients, with relative steepness changing two-fold from one to another, and designate them as N0, N1 and N2. The corresponding gradient functions are
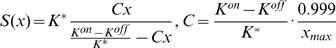
(12)with 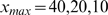 mm for N0, N1 and N2, respectively. Here 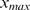 is the size of square 2D domain, where cells were simulated starting from the center of left wall 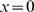.
